# Late-onset riboflavin-responsive multiple acyl-CoA dehydrogenase deficiency (MADD): case reports and epidemiology of ETFDH gene mutations

**DOI:** 10.1186/s12883-019-1562-5

**Published:** 2019-12-18

**Authors:** Wei Chen, Youqiao Zhang, Yifeng Ni, Shaoyu Cai, Xin Zheng, Frank L. Mastaglia, Jingshan Wu

**Affiliations:** 1 Department of Neurology, The Second Affiliated Hospital of Shantou University Medical College, Shantou, Guangdong China; 2 Perron Institute for Neurological and Translational Science, QE II Medical Centre, 8 Verdun Street, Nedlands, Western Australia Australia; 3 Faculty of Health and Medical Sciences, The University of Western Australia, (M503), 35 Stirling Highway, Perth, Western Australia 6009 Australia

**Keywords:** Late-onset multiple acyl-CoA dehydrogenase deficiency, Lipid storage myopathy, ETFDH gene, C.250G > A mutation, Southern min population, Epidemiology

## Abstract

**Background:**

Multiple acyl-CoA dehydrogenase deficiency (MADD) is a riboflavin-responsive lipid-storage myopathy caused by mutations in the EFTA, EFTB or ETFDH genes. We report a Chinese family of Southern Min origin with two affected siblings with late-onset riboflavin-responsive MADD due to a homozygous c.250G > A EFTDH mutation and review the genetic epidemiology of the c.250G > A mutation.

**Case presentation:**

Both siblings presented with exercise-induced myalgia, progressive proximal muscle weakness and high levels of serum muscle enzymes and were initially diagnosed as polymyositis after a muscle biopsy. A repeat biopsy in one sibling subsequently showed features of lipid storage myopathy and genetic analysis identified a homozygous mutation (c.250G > A) in the ETFDH gene in both siblings and carriage of the same mutation by both parents. Glucocorticoid therapy led to improvement in muscle enzyme levels, but little change in muscle symptoms, and only after treatment with riboflavin was there marked improvement in exercise tolerance and muscle strength. The frequency and geographic distribution of the c.250G > A mutation were determined from a literature search for all previously reported cases of MADD with documented mutations. Our study found the c.250G > A mutation is the most common EFTDH mutation in riboflavin-responsive MADD (RR-MADD) and is most prevalent in China and South-East Asia where its epidemiology correlates with the distribution and migration patterns of the southern Min population in Southern China and neighbouring countries.

**Conclusions:**

Mutations in ETFDH should be screened for in individuals with lipid-storage myopathy to identify patients who are responsive to riboflavin. The c.250G > A mutation should be suspected particularly in individuals of southern Min Chinese background.

## Background

Multiple acyl-CoA dehydrogenase deficiency (MADD) is an autosomal recessive disorder caused by mutation of the electron transfer flavoprotein A (ETFA), electron transfer flavoprotein B (ETFB), or electron transfer flavoprotein dehydrogenase (ETFDH) genes, resulting in dysfunction of mitochondrial electron transfer and lipid storage myopathy (LSM) [1–3]. MADD is classified into a severe early-onset form and a milder late-onset form manifesting in adolescence or adult-life with fluctuating exercise intolerance, myalgia, progressive proximal muscle weakness, and in some cases hypoglycaemia and metabolic acidosis. Most MADD patients improve dramatically after treatment with riboflavin, thus the condition is called riboflavin-responsive MADD (RR-MADD). The late-onset form accounts for about 90% of cases of LSM in China and is related to ETFDH deficiency [1, 4]. A wide spectrum of different ETFDH mutations has been reported worldwide, of which c.250G > A (p.Ala84Thr) is the most common, being found predominantly in southern China, while c.770A > G (p.Try257Cys) and c.1227A > C (p.Leu409Phe) are more common in northern China [4].

Here we report two brothers with adolescent RR-MADD from a southern Min Chinese pedigree who were homozygous for the c.250G > A mutation, and were initially misdiagnosed as having an inflammatory myopathy. We also review the genetic epidemiology of the c.250G > A mutation in China and around the world which is strongly suggestive of a founder effect in the southern Min Chinese population.

## Case presentation

### Case 1

The proband was a 19-year-old male with a 3 month history of exercise intolerance, myalgia and muscle weakness which was more severe in the lower limbs. Physical examination found reduced muscle tone and mild weakness of proximal muscles (MRC 4/5). Electromyography revealed myopathic motor unit changes in limb muscles without spontaneous discharges. A presumptive diagnosis of polymyositis was made and he was started on prednisone 1 mg/kg for 3 weeks, during which the serum creatine kinase (CK) level fell from 911 U/L to 190 U/L (normal range 0-174 U/L) and there was slight improvement in muscle weakness, but the myalgia and exercise intolerance persisted. Levels of other biochemical parameters also dropped but still remained above the normal range, including LDH (1624 U/L to 1066 U/L), AST (188 U/L to 76 U/L) and uric acid (738 μmol/L to 665 μmol/L) after treatment with prednisone.

### Case 2

A 13-year-old male, the younger brother of the proband, presented with a 1 year history of progressive muscle weakness and myalgia worse after exercise. Neurological examination found reduced muscle tone and mild proximal weakness in the lower limbs (MMT 4/5). Biochemical tests showed raised serum CK (2165 U/L), CK-MB (103 ng/ml) and uric acid levels (709 μmol/L). MRI of the lower limbs showed focal areas of increased signal intensity in the gluteal and thigh muscles on short time inversion recovery (STIR) sequences, suggesting a diagnosis of possible polymyositis. A muscle biopsy taken from the right biceps femoris showed only a small focal lymphocytic infiltrate but no other abnormalities. Echocardiography showed mildly reduced ventricular diastolic function. He was commenced on oral prednisone (1 mg/kg), following which there was a fall in the serum CK (2165 U/L to 612 U/L), CK-MB (103 ng/ml to 51 ng/ml) and uric acid level (709 μmol/L to 415 μmol/L), but there was no improvement in his muscle symptoms.

The patient then reported that he had an elder brother (the proband) who had similar symptoms, and that their parents did not have any muscle symptoms and were not consanguineous. A second muscle biopsy from the left quadriceps femoris did not provide any confirmatory evidence of an inflammatory myopathy but revealed prominent cytoplasmic vacuolation of muscle fibres strongly suggestive of a lipid storage myopathy. The patient was then started on riboflavin (30 mg, three times per day) and the prednisone was discontinued. After 3 days of riboflavin therapy there was already considerable improvement in muscle strength and exercise tolerance.

**Fig. 1:**
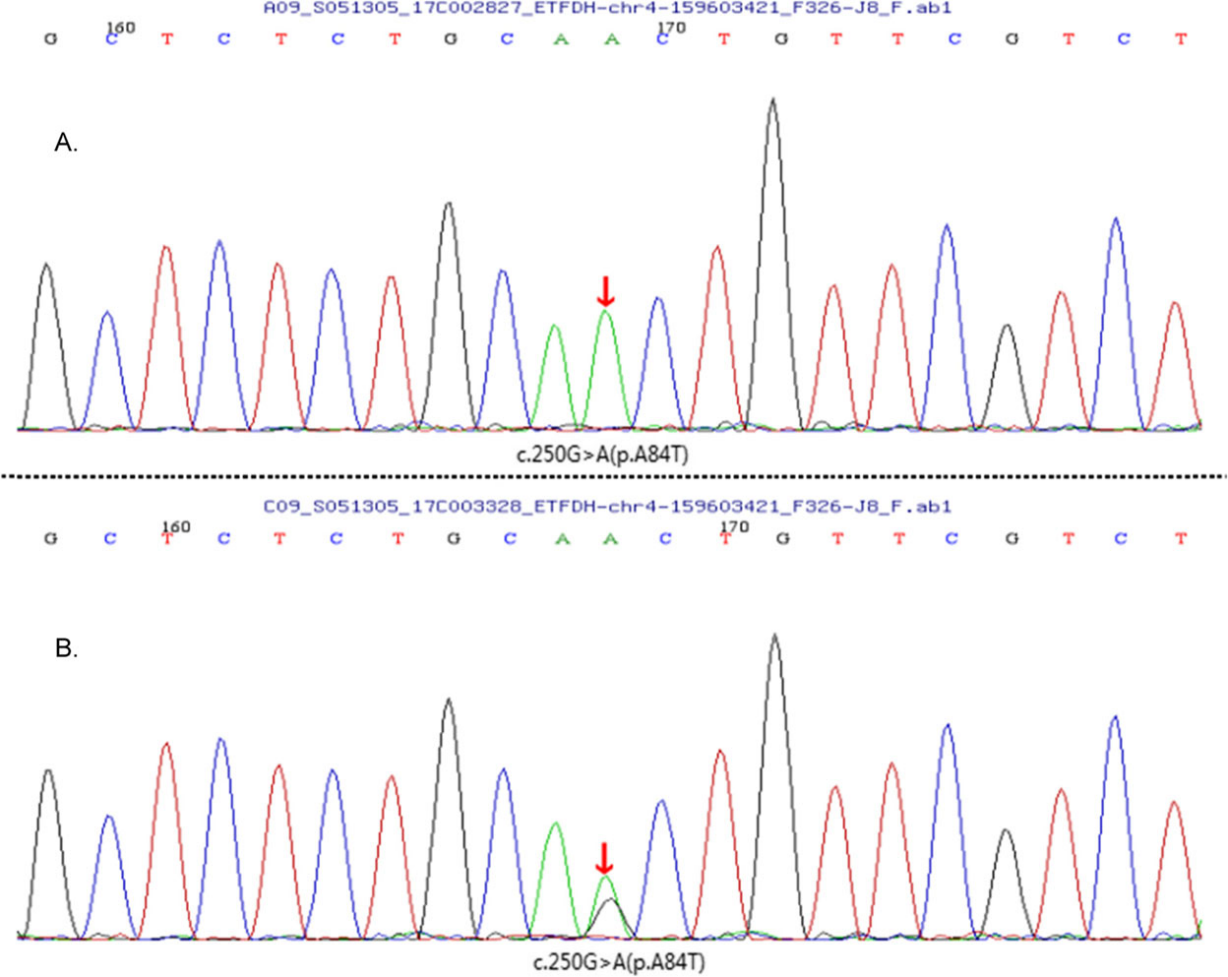
Sequencing of the electron transfer flavoprotein dehydrogenase gene of the two patients (**a**) and their parents (**b**), showed the same missense mutation of c.250G > A (p.A84T) in exon 3, chr4–159,603,421. In patient 1 and patient 2, the mutation was homozygous while in their parents it was heterozygous

Late-onset MADD was suspected and blood was collected from all family members and sent to MyGenostics for genetic analysis. Sanger sequencing was performed for the ETFA, ETFB and ETFDH genes as well as other neuromuscular disease related genes. This identified a homozygous ETFDH gene mutation of c.250G > A (p.A84T) in exon 3, chr4–159,603,421 (Fig.1a) in both brothers, and a heterozygous mutation in both parents (Fig.1b). No variants were detected in ETFA, ETFB or other genes.

After 1 month of riboflavin treatment there was marked improvement in myalgia and exercise intolerance in both brothers and the MMT scores in proximal limb muscles had improved to 5/5. Both patients have since been able to walk and run freely and the younger brother is now able to participate in school sporting activities. Serum CK levels fell markedly after commencement of riboflavin and have remained normal in both patients over the past year.

### Genetic epidemiology of the ETFDH c.250G > A mutation

We ascertained the frequency of reported cases of MADD with confirmed ETFDH mutations through Pubmed and the Wanfang Database in China. In the reviews of 350 late-onset cases of MADD by Grunert in 2014 and 90 cases by Xi in 2013 [5], 244 individuals had confirmed EFTDH mutations. A further 137 cases of EFTDH mutation related MADD have since been reported and were added into our study. In total, there are 381 cases of MADD with 113 different EFTDH mutations reported in over 110 studies to date [5–44] (Additional file 1: Table S1). The frequencies of the 6 most common mutations are shown in Table 1. Overall, the c.250G > A mutation is the most common ETFDH mutation, accounting for 28.1% of reported cases, 59 of which were homozygous (including our cases) and 48 cases had compound heterozygous mutations. The other common mutations were c.770A > G (12.9%) and c.1227A > C (8.9%) in Chinese, and c.1130 T > C (6.3%) in the Turkish population [37].

**Table 1:** The frequency and ethnic distribution of the 6 most common ETFDH mutations among 381 reported cases of MADD with a proven ETFDH mutation

ETFDH	Homozygous	Heterozygous	Population	Total cases	Percentage
c.250G > A	59	48	Chinese/Asian	107	28.1%
c.770A > G	4	45	Chinese/Asian	49	12.9%
c.1227A > C	1	33	Chinese	34	8.9%
c.1130 T > C	24	0	Turkish	24	6.3%
c.389 A > T	0	22	Chinese	22	5.8%
c.1367C > T	4	5	Caucasians/Japanese	9	2.4%

**Fig. 2:**
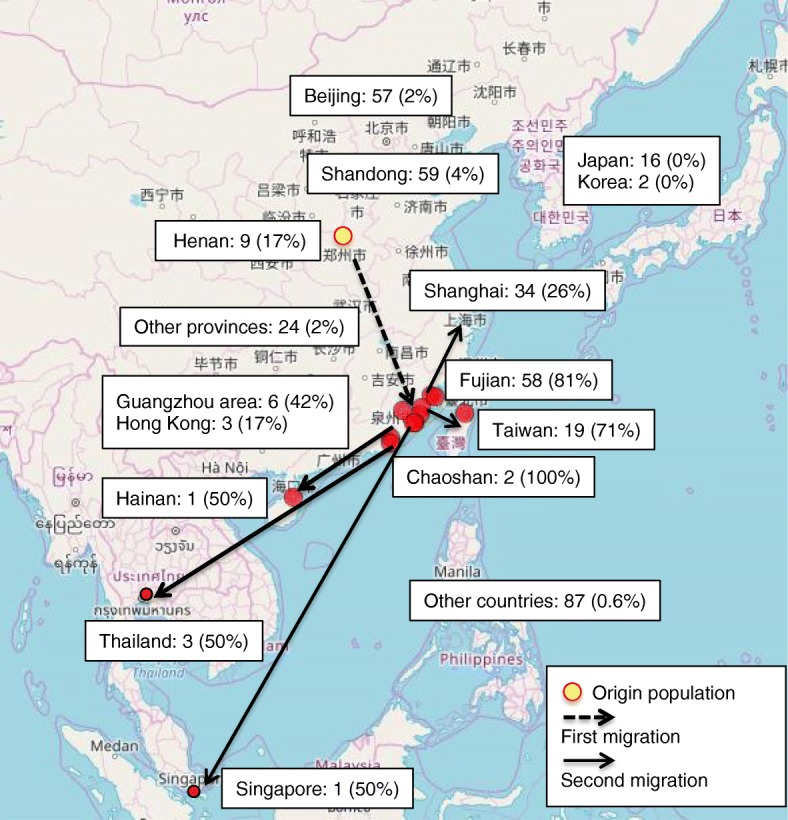
Genetic epidemiology of the c.250G > A ETFDH mutation. Geographic distribution of the Southern Min population (highlighted in red); reported number of MADD case with confirmed ETFDH mutation; and the allele frequency of the c.250G > A ETFDH mutation among all reported ETFDH mutations in different regions of China and neighboring countries. The Southern Min people is believed to have migrated from middle China around Henan Province (origin) to the Southern Min region (first migration) over a thousand years ago, and further travelled to Southeast Asia and overseas in a second migration in the past centuries. Map downloaded and modified from Glottolog 4.1 edited by HMS, H&F, R& HPM, M/ CC BY https://glottolog.org/resource/languoid/id/minn1248.bigmap.html#3/23.63/115.88

We further analysed the distribution of the EFTDH mutation cases and the frequency of the c.250G > A allele among all reported EFTDH mutations in different Chinese provinces/regions, South-East Asian countries, and globally. As shown in Fig. 2, the highest frequencies of c.250G > A mutation MADD cases were observed in the Fujian, Guangzhou and Chaoshan regions, and in Taiwan and Hainan islands, all of which have a strong southern Min background. We estimated the approximate net Southern Min population in each province/region in China and neighbouring countries, and the approximate percentage of the population they comprise based on data from the Internet. As showed in Table 2, the percentage of c.250G > A allele is significantly correlated with the distribution of southern Min population in China and surrounding countries (Spearman correlation *p* < 0.01), suggesting a founder effect of the c.250G > A mutation in this population.

**Table 2:** Correlation of genetic epidemiology of the ETFDH C.250G > A mutation and Southern Min Population

Regions and Countries	Southern Min (%)*	SouthernMin (millions)	No. ETFDH MADD cases	c.250G > A mutation (%)*	c.250G > A alleles (No.)	Homozygous cases	Heterozygous cases
Henan	Origin	–	9	17%	3	0	3
Chaoshan^1^	~ 90%	~ 14 m	2	100%	4	2	0
Fujian^1,3^	~ 60%	~ 20 m	58	81%	94	40	14
Taiwan^2,3^	~ 70%	~ 15.6 m	19	71%	27	10	7
Hainan^1,3^	~ 59%	~ 5 m	1	50%	1	0	1
Singapore^2,3^	~ 37%	~ 1.86 m	1	50%	1	0	1
Guangzhou area^1,3^	~ 6%	~ 3 m	6	42%	5	1	3
Hong Kong^2,3^	~ 6.7%	~ 0.5 m	3	17%	1	0	1
Thailand^2,3^	~ 9%	~ 5 m	3	50%	3	0	3
Shanghai area^a 1,3^	~ 1%	~ 2 m	34	26%	18	6	6
Shandong^a^	< 1%	–	59	4%	5	0	5
Beijing^a^	< 1%	–	57	2%	2	0	2
Other provinces	< 1%	–	24	2%	1	0	1
Japan	< 1%	–	16	0%	0	0	0
Korea	< 1%	–	2	0%	0	0	0
Other Countries^1,2,3^	< 1%	~ 20 m	87	0.6%	1	0	1
Total			381		169	59	48

*There is significant correlation of the estimated percentage of Southern Min population and the percentage of c.250G > A in total ETFDH mutation cases (Spearman correlation coefficient, *p* < 0.01)

c.250G > A mutation (%): The allele frequency of the c.250G > A ETFDH mutation among all the reported cases in the regions/countries, assumed two ETFDH mutation alleles in each case

^a^Major Neuromuscular centres in China

Guangzhou area: including Guangzhou and surrounding cities

Shanghai area: including Shanghai and surrounding cities

m: millions

Estimated net number and percentage of Southern Min population in the regions calculated based on local population reports from Internet

Data recourse: 1 Baidu; 2 Ethnologue; 3 Wikipedia,

Three cases of MADD with the c.250G > A mutation have been reported in Thailand, where over 10% of the population is of Chinese descent [7], the majority of them being southern Min people who migrated from the Chaoshan region of China in the last two centuries. The only reported case of MADD with the c.250G > A mutation in Western countries is in France, in an individual of Chinese origin [35]. There have not been any reported cases with the c.250G > A mutation among Koreans [13], Japanese [45] or Caucasians.

## Discussion

We report a Southern Min Chinese family with two siblings carrying the c.250G > A (p.Ala84Thr) mutation in the ETFDH gene who presented with typical features of adolescent-onset riboflavin responsive multiple acyl-CoA dehydrogenase deficiency (MADD) associated lipid-storage myopathy. In addition, Case 2 also had echocardiographic evidence of ventricular diastolic dysfunction and cardiomyopathy, which has been commonly reported in early-onset MADD, but is rare in the late-onset form of the disease [1, 4, 46–49]. As late-onset MADD is a rare disease and the symptoms are nonspecific, it is not uncommon for the diagnosis to be delayed and for the condition to be misdiagnosed, as occurred in the present cases who were initially thought to be suffering from an inflammatory myopathy [4, 46–48]. Although less effective than riboflavin, glucocorticoid therapy may induce short-term symptomatic improvement in some cases of late-onset MADD, which may also suggest a diagnosis of an inflammatory myopathy and may result in continued administration of glucocorticoids and associated steroid side-effects [4, 50]. Our cases underline the importance of obtaining a detailed family history at the time of presentation in such cases, as well as the potential pitfalls of making a diagnosis of inflammatory myopathy on clinical grounds alone, or in the absence of conclusive muscle biopsy evidence of an inflammatory myopathy.

Our cases are the first reported from the Chaoshan region in Guangdong province, which is geographically in close proximity to Fujian and Taiwan, where the majority of c.250G > A ETFDH mutations have been reported [15, 47, 48]. A total of 52 cases with homozygous c.250G > A mutations have been reported in these regions, where the carrier frequency of c.250G > A has been estimated to be 1.35% [4, 15, 47, 51]. People from these regions share a similar ethnic background and speak the southern Min (Min Nan) dialect. The southern Min people are believed to have migrated from middle China around Henan Province to the remote and isolated southern Min region over a thousand years ago (first migration), and further travelled to Southeast Asia and overseas in a second migration in past centuries and account for the majority of immigrant Chinese in South-East Asian countries. Although the previous report by Xi et al. suggested that the c.250G > A mutation is more common in Southern China, south of the Qin Mountains [4], the Henan province located in central China, which is historically the origin of Southern Min population has an allele frequency of 17% of c.250G > A mutation among 9 reported cases of MADD [6, 18, 20, 52]. In contrast, a report from Jiangxi province located in South China geographically next to Fujian found no such mutation in 13 MADD cases [38]. As shown in Fig. 2, the geographic distribution of the c.250G > A mutation is mostly consistent with the distribution and migration route of the southern Min Chinese population in China and Southeast Asia, and strongly suggests a founder effect in this population.

## Conclusion

These cases emphasize the importance of considering the diagnosis of a lipid storage myopathy and RR-MADD in patients presenting with muscle weakness, myalgia and exercise intolerance, and of screening for mutations in the ETFDH gene to identify patients who are responsive to riboflavin therapy. In particular, the diagnosis of RR-MADD and screening for the c.250G > A ETFDH mutation should be considered in Chinese individuals of southern Min background who present in this way, whether they are in China or have migrated to other countries.

## Supplementary information

**Additional file 1: Table S1.** 381 cases of MADD with confirmed ETFDH mutation in the literature from Pubmed and Wanfang database.

## Data Availability

Please see supplement materials.

## Abbreviations

AST: Aspartate transaminase
CK: Creatine kinase
CK-MB: Creatine kinase-muscle/brain
ETFA: Electron transfer flavoprotein A
ETFB: Electron transfer flavoprotein B
ETFDH: Electron transfer flavoprotein dehydrogenase
LSM: Lipid storage myopathy
MADD: Multiple acyl-CoA dehydrogenase deficiency
MMT: Manual Muscle Testing
MRI: Magnetic resonance imaging
RR-MADD: Riboflavin-responsive Multiple acyl-CoA dehydrogenase deficiency
STIR: Short time inversion recovery
