# Supplementary material for: Late-onset riboflavin-responsive multiple acyl-CoA dehydrogenase deficiency (MADD): case reports and epidemiology of ETFDH gene mutations
Source: BMC Neurol. 2019 Dec 18;19:330. doi: 10.1186/s12883-019-1562-5 (PMC6921586; doi:10.1186/s12883-019-1562-5)
Supplement: Supplementary file 1 — Additional file 1: Table S1. 381 cases of MADD with confirmed ETFDH mutation in the literature from Pubmed and Wanfang database. [file 12883_2019_1562_MOESM1_ESM.docx]

Supplemental table. 381 cases of MADD with confirmed ETFDH mutation in the literature from Pubmed and Wanfang database.

| Sub  total | | # | Region/Country | | ETFDH | | Age of onset,  Gender | Author year |
| --- | --- | --- | --- | --- | --- | --- | --- | --- |
|  |  |  |  |  | Nucleotide change  allele 1  allele 2 | Amino acid change  (predicted from c.DNA) |  |  |
| 9 | | 1 | Henan | | c.1204A>T;  c.684+1G>T | p.T402S, | 13y, F | Xu 2019 [1] |
|  |  | 2 | Henan | | c.1395dupT  c.770A＞G | p.G466W  p.Y257C | 38y, F | Chen 2018 [2] |
|  |  | 3 |  |  | c.770A＞G  c.770A＞G | p.Y257C  p.Y257C | 18y, M |  |
|  |  | 4 |  |  | c.770A＞G  c.770A＞G | p.Y257C  p.Y257C | 16y, M |  |
|  |  | 5 | Henan | | c.250G>A,  c.1691-3C>G | p.A84T | 5y, M | Cui 2017 [3] |
|  |  | 6 | Henan | | c.814 G >A  c.389 A>T | p.D130V | 34y, F | Chen 2018 [4] |
|  |  | 7 | Henan | | c.250G>A  c.498G>A | p.A84T  p.M166I | 11y, M (twin) | Zhen 2019 [5] |
|  |  | 8 |  |  | c.250G>A  c.498G>A | p.A84T  p.M166I | 11y, F  (twin) |  |
|  |  | 9 | Henan | | c.389A N T  c.736G N A | p. D130V  p. E246K | 9y, F | Zhuo, 2015 [6] |
| 2 | | 10 | Chaoshan | | c.250G>A  c.250G>A | p.A84T  p.A84T | 19y, M | Present study |
|  |  | 11 |  |  | c.250G>A  c.250G>A | p p.A84T  p.A84T | 12y, M |  |
| 58 | | 12 | Fujian | | c.250G>A  c.892C > T | p.A84T  p.P298S | 23y, M | Fu 2016 [7] |
|  |  | 13 |  |  | c.250G>A  c.453delA  c.449_453delTAACA | p.A84T  p.G52AT15 p.L150T | 15y, M |  |
|  |  | 14 |  |  | c.250G>A  c.453delA  c.449_453delTAACA | p.A84T  p.G152AfsT15 p.L150T | 68y, M |  |
|  |  | 15 | Fujian | | c.250G>A  c.250G>A | p.A84T  p.A84T | 46yo, M | Chen 2019 [8] |
|  |  | 16 |  |  | c.250G>A  c.959C>T | p.A84T | 60yo, M |  |
|  |  | 17-55 | Fujian  39 cases | | c.250G>A  c.250G>A | p.A84T  p.A84T | 24 F,  32M  Mean 24.5+12.6 y  (4-55y) | Wang 2011 [9] |
|  |  | 56-61 | Fujian  6 cases | | c.250G>A  c.524G>A | p.A84T  p.A175H |  |  |
|  | 62-63 | Fujian  2 cases | | c.250G>A  c.643G>A | p.A84T  p.A215T |  |  |  |
|  | 64-65 | Fujian  2 cases | | c.250G>A  c.770A>G | p.A84T  p.T257C |  |  |  |
|  | 66 | Fujian | | c.250G>A  c.998A>G | p.A84T  p.T333C |  |  |  |
|  | 67 | Fujian | | c.524G>A  c.770A>G | p.A175H  p.T257C |  |  |  |
|  | 68 | Fujian | | c.770A>G  c.1254-1257del | p.T257C  p. L418TfsX10 |  |  |  |
|  | 69 | Fujian | | c.1395T>G | p.T465X |  |  |  |
| 19 | 70 | Taiwan | | c.250G > A  c.383T > C | p.A84T  p.F128S | 9y, F | Er 2011 [10]  Chien 2013 [11] |  |
|  | 71 | Taiwan | | c.250G>A  c.250G>A | p.A84T  p.A84T | 43y, M | Lan 2010 [12] |  |
|  | 72 |  |  | c.250G>A  c.250G>A | p.A84T  p.A84T | 34y, F |  |  |
|  | 73 |  |  | c.250G>A  c.250G>A | p.A84T  p.A84T | 21y, M |  |  |
|  | 74 |  |  | c.250G>A  c.250G>A | p.A84T  p.A84T | 29y, F |  |  |
|  | 75 |  |  | c.250G>A  c.250G>A | p.A84T  p.A84T | 19y, F |  |  |
|  | 76 |  |  | c.250G>A  c.250G>A | p.A84T  p.A84T | 7y, F |  |  |
|  | 77 |  |  | c.250G>A  c.524G>A  c.863C>G | p.A84T  p.R175H  p.S288W | 20y, M |  |  |
|  | 78 | Taiwan | | c.250G>A  c.1831GNA | p.A84T  p.G611R | 10y, M | Er 2010 [10] |  |
|  | 79 |  |  | c.250G>A  c.1831GNA | p.A84T  p.G611R | 8y, F |  |  |
|  | 80 | Taiwan | | c.250G>A  c.250G>A | p.A84T  p.A84T | 11y, F | Er 2010 [10] |  |
|  | 81 |  |  | c.250G>A  c.250G>A | p.A84T  p.A84T | 12y, M |  |  |
|  | 82 |  |  | c.250G>A  c.250G>A | p.A84T  p.A84T | 21y, M |  |  |
|  | 83 | Taiwan | | c.250G>A  c.524G>T | p.A84T  p.R175L | Early childhood , F | Er 2010 [10]  Liang 2009 [13] |  |
|  | 84 |  |  | c.250G>A  c.524G>T | p.A84T  p.R175L | 12y, F |  |  |
|  | 85 |  |  | c.250G>A  c.250G>A | p.A84T  p.A84T | 14y, M |  |  |
|  | 86 |  |  | c.250G>A  c.380T>A | p.A84T  p.127H | 10y, F |  |  |
|  | 87 | Taiwan | | c.251C>T  c.251C>T | p.A84V  p.A84V | 17y, n.a | Chien 2013 [11] |  |
|  | 88 |  |  | c.295C>G | p.R99G | -,F |  |  |
| 1 | 89 | Hainan | | c.250G>A  c.380T>C | p.A84T | 6y, M | Lu 2014 [14] |  |
| 1 | 90 | Singapore | | c.250G > A  c.770A > G  c.1042C > T | p.Ala84Thr  p.Tyr257Cys  p.Arg348Trp | 17y, M | Goh 2018 [15] |  |
| **6** | 91 | Guangzhou area,  Guangzhou | | c.250G>A  c.250G>A | Ala84Thr  Ala84Thr | 13y, F | Cao 2014 [16] |  |
|  | 92 |  |  | c.250G > A  c.524G > A | Ala84Thr Arg175His | 14y, M |  |  |
|  | 93 |  |  | c.250G > A  c.524G > A | Ala84Thr Arg175His | 14y, M |  |  |
|  | 94 | Guangzhou area Hui Zhou | | c. 770A > G  c. 1270_1273del | p.Y257C | 71y, F | Kong 2018 [17] |  |
|  | 95 |  |  | c. 770A > G  c. 1270_1273del | p.Y257C | 70y, F |  |  |
|  | 96 | Guangzhou area Shenzhen | | c.250G>A  c.353G>T | p.Ala84Thr  p.Cys118Phe | 13y, F | Dai 2016 [18] |  |
| 3 | 97 | Hong Kong | | c.1355delG  c.250G>A | p.R452Kfs*3  p.A84T | 3 y ,F | Law 2009 [19] |  |
|  | 98 |  |  | c.409CNT  c.1400GNC | p.P137S  p.G467R | 10 mo, F |  |  |
|  | 99 |  |  | c.409CNT  c.1400GNC | p.P137S  p.G467R | 18 mo, F |  |  |
| 3 | 100 | Thailand | | c.250G>A  c.524G>A | p.A84T  Arg175His | 40 y ,F | Santananukarn , 2019 [20] |  |
|  | 101 | Thailand | | c.250G>A  c.832-1G>A | p.A84T | 9 y, M | Wasant 2010 [21] |  |
|  | 102 |  |  | c.250G>A  c.832-1G>A | p.A84T | 7 y, M |  |  |
| 34 | 103 | Shanghai area | | c.250G > A  c.920C > G | p.A84T  p.S307C | 23 y, F | Xue, 2017[22] |  |
|  | 104 | Shanghai area | | c.250G>A  c.250G>A | p.A84T  p.A84T | Mean age of onset  25.0±13.3 years old (4-  36 years)  38 females  52 males. | Xi 2013[23] |  |
|  | 105 |  | | c.250G>A  c.250G>A | p.A84T  p.A84T |  |  |  |
|  | 106 |  |  | c.1227A>C  c.1227A>C | p.L409F  p.L409F |  |  |  |
|  | 107 |  |  | c.250G>A  c.250G>A | p.A84T  p.A84T |  |  |  |
|  | 108 |  |  | c.191G>A  c.191G>A | p.R64K  p.R64K |  |  |  |
|  | 109 |  |  | c.250G>A  c.250G>A | p.A84T  p.A84T |  |  |  |
|  | 110 |  |  | c.250G>A  c.250G>A | p.A84T  p.A84T |  |  |  |
|  | 111 |  |  | c.1454C>G  c.1454C>G | p.T485S  p.T485S |  |  |  |
|  | 112 |  | | c.250G>A  c.250G>A | p.A84T  p.A84T |  |  |  |
|  | 113 |  |  | c.770A>G  c.1378G>T | p.Y257C  p.G460* |  |  |  |
|  | 114 |  |  | c.409C>T  c.1448C>T | p.P137S  p.P483L |  |  |  |
|  | 115 |  |  | c.433G>C  c.949C>A # | p.D145H  p.P317T |  |  |  |
|  | 116 |  |  | c.770A>G  c.1763A>G | p.Y257C  p.H588R |  |  |  |
|  | 117 |  |  | c.1212T>C  c.1227A>C | p.M404T  p.L409F |  |  |  |
|  | 118 |  |  | c.389A>T  c.872T>G | p.D130V  p.V291G |  |  |  |
|  | 119 |  |  | c.405+1G>T  c.770A>G | Truncated  p.Y257C |  |  |  |
|  | 120 |  |  | c.250G>A  c.998A>G | p.A84T  p.Y333C |  |  |  |
|  | 121 |  |  | c.524G>T  c.770A>G | p.R175L  p.Y257C |  |  |  |
|  | 122 |  |  | c.226G>A；  c.227C>A  c.250G>A | p.A76K  p.A84T |  |  |  |
|  | 123 |  |  | c.389A>T  c.1084G>A | p.D130V  p.G362R |  |  |  |
|  | 124 |  |  | c.770A>G  c.1395T>G | p.Y257C  p.Y465* |  |  |  |
|  | 125 |  |  | c.1281_1282del  c.1227A>C | p.I428Rfs*6  p.L409F |  |  |  |
|  | 126 |  |  | c.172G>A#  c.1454C>G | p.E58K  p.T485S |  |  |  |
|  | 127 |  |  | c.1395T>G  c.1744A>T | p.Y465*  p.N582Y |  |  |  |
|  | 128 |  |  | c.250G>A  c.1227A>C | p.A84T  p.L409F |  |  |  |
|  | 129 |  |  | c.250G>A  c.976G>C | p.A84T  p.G326R |  |  |  |
|  | 130 |  |  | c.3G>C  c.770A>G | p.M1?  p.Y257C |  |  |  |
|  | 131 |  |  | c.349G>C  c.1227A>C | p.A117P  p.L409F |  |  |  |
|  | 132 |  |  | c.770A>G  c.1691-3C>G | p.Y257C  truncated |  |  |  |
|  | 133 |  |  | c.1454C>G | p.T485S |  |  |  |
|  | 134 |  |  | c.1212T>C | p.M404T |  |  |  |
|  | 135 |  |  | c.152G>A | p.R51Q |  |  |  |
|  | 136 |  |  | c.1395T>G | p.Y465* |  |  |  |
| 59 | 137 | Shandong | | c.1211T>C  c.1450 T>C | p.M404T  p.W484R | 24 y, M | Wen, 2015 [24] |  |
|  | 138 | Shandong | | c.770A>G  c.770A>G  c.993T>G | p.Y257C  p.Y257C  p.N331K | The average age of onset  of disease was 25.0±13.3 years old, with a range of 4-  36 years, and the gender ratio was about 1:1.4 (38 females  vs. 52 males). | Xi 2013 [25] |  |
|  | 139 |  |  | c.1227A>C  c.872T>G | p.L409F  p.V291G |  |  |  |
|  | 140 |  |  | c.1227A>C  c.872T>G | p.L409F  p.V291G |  |  |  |
|  | 141 |  |  | c.1227A>C  c.393G>C | p.L409F  p.W131C |  |  |  |
|  | 142 |  |  | c.1227A>C  c.1399G>C | p.L409F  p.G467R |  |  |  |
|  | 143 |  |  | c.242T>C  c.487-9T>C | p.L81P  truncated |  |  |  |
|  | 144 |  |  | c.1295T>A  c.1528C>T | p.V432G  p.P510S |  |  |  |
|  | 145 |  |  | c.1227A>C  c.1691-3C>G | p.L409F  truncated |  |  |  |
|  | 146 |  |  | c.380T>G  c.1531G>A | p.L127R  p.D511N |  |  |  |
|  | 147 |  |  | c.770A>G  c.872T>G | p.Y257C  p.V291G |  |  |  |
|  | 148 |  |  | c.770A>G  c.973del312 | p.Y257C  p.325del48 |  |  |  |
|  | 149 |  |  | c.770A>G  c.1084G>A  c.1399G>A | p.Y257C  p.G362R  p.G467R |  |  |  |
|  | 150 |  |  | c.1227A>C  c. 389A>T | p.L409F  p.D130V |  |  |  |
|  | 151 |  |  | c.1436G>C  c.1395T>G | p.R479T  p.Y465* |  |  |  |
|  | 152 |  | | c.715G>A  c.1395T>G | p.A239T  p.Y465* |  |  |  |
|  | 153 |  |  | c.1227A>C  c.872T>G | p.L409F  p.V291G |  |  |  |
|  | 154 |  |  | c.770A>G  c.1448C>T | p.Y257C  p.P483L |  |  |  |
|  | 155 |  |  | c.1227A>C  c.503A>G | p.L409F  p.N168S |  |  |  |
|  | 156 |  |  | c.1227A>C  c.692T>C | p.L409F  p.F231S |  |  |  |
|  | 157 |  |  | c.770A>G  c.1395T>G | p.Y257C  p.Y465* |  |  |  |
|  | 158 |  |  | c.1227A>C  c.524G>T | p.L409F  p.R175L |  |  |  |
|  | 159 |  |  | c.770A>G  c.1099A>G | p.Y257C  p.N367D |  |  |  |
|  | 160 |  |  | c.389A>T  c.361_362 insT | p.D130V  p.P121Lfs*5 |  |  |  |
|  | 161 |  |  | c.1227A>C  c.389A>T | p.L409F  p.D130V |  |  |  |
|  | 162 |  |  | c.236C>G  c.1281_1282del | p.A79G  p.I428Rfs*6 |  |  |  |
|  | 163 |  |  | c.389A>T  c.835T>C | p.D130V  p.W279R |  |  |  |
|  | 164 |  |  | c.353G>T  c.1657T>C | p.C118F  p.Y553H |  |  |  |
|  | 165 |  |  | c.1227A>C  c.213_215del | p.L409F  p.V72del |  |  |  |
|  | 166 |  |  | c.770A>G  c.1281_1282del | p.Y257C  p.I428Rfs*6 |  |  |  |
|  | 167 |  |  | c.389A>T  c.835T>C | p.D130V  p.W279R |  |  |  |
|  | 168 |  |  | c.770A>G  c. -75A>G | p.Y257C  Transcript↓ |  |  |  |
|  | 169 |  |  | c.770A>G  c.1531G>A  c.250G>A | p.Y257C  p.D511N  p.A84T |  |  |  |
|  | 170 |  |  | c.389A>T  c.1395T>G | p.D130V  p.Y465* |  |  |  |
|  | 171 |  |  | c.1227A>C  c.250G>A | p.L409F  p.A84T |  |  |  |
|  | 172 |  |  | c.770A>G  c.872T>G | p.Y257C  p.V291G |  |  |  |
|  | 173 |  |  | c.770A>G  c.1372_1375del | p.Y257C  p.C458Tfs*10 |  |  |  |
|  | 174 |  |  | c.250G>A  c.524G>A | p.A84T  p.R175H |  |  |  |
|  | 175 |  |  | c.256C>T  c.175+2T>C | p.R86C  Truncated |  |  |  |
|  | 176 |  |  | c.770A>G  c.1099A>G | p.Y257C  p.N367D |  |  |  |
|  | 177 |  |  | c.250G>A  c.528G>C | p.A84T  p.L176F |  |  |  |
|  | 178 |  |  | c.770A>G  c.1027T>C | p.Y257C  p.W343R |  |  |  |
|  | 179 |  |  | c.770A>G  c.1026G>T | p.Y257C  p.R342S |  |  |  |
|  | 180 |  |  | c.242T>C  c. 606+7A>G | p.L81P  Truncated |  |  |  |
|  | 181 |  |  | c.389A>T  c.1395T>G | p.D130V  p.Y465* |  |  |  |
|  | 182 |  |  | c.389A>T  c.503A>G | p.D130V  p.N168S |  |  |  |
|  | 183 |  |  | c.389A>T  c.1205C>T | p.D130V  p.T402I |  |  |  |
|  | 184 |  |  | c.770A>G  c.1448C>T | p.Y257C  p.P483L |  |  |  |
|  | 185 |  |  | c.250G>A  c.389A>T | p.A84T  p.D130V |  |  |  |
|  | 186 |  |  | c.770A>G | p.Y257C |  |  |  |
|  | 187 |  |  | c.1212T>C | p.M404T |  |  |  |
|  | 188 |  |  | c.1227A>C | p.L409F |  |  |  |
|  | 189 |  |  | c.251C>T | p.A84V |  |  |  |
|  | 190 |  |  | c.1227A>C | p.L409F |  |  |  |
|  | 191 |  |  | c.389A>T | p.D130V |  |  |  |
|  | 192 |  |  | c.770A>G | p.Y257C |  |  |  |
|  | 193 |  |  | c.770A>G | p.Y257C |  |  |  |
|  | 194 |  | | c. 405+1G>A | Truncated |  |  |  |
|  | 195 | Shandong | | c.1522C>A  c.1583_1584insA | p.P508T  p.N528Kfs*3 | 45 y, M | Zhao 2012 [26] |  |
| 57 | 196 | Beijing | | c.770A＞ G  c.1281-1282AA | p.Y257C  p.I428 Rfs6 | 1d, M | Hu 2018[27] |  |
|  | 197 | Beijing | | c.265-266delCA  c.1211 T > C |  | 22 y, M | Hong 2018[28] |  |
|  | 198 |  |  | c.34G > C  c.736G > A | p.A12P  p.E246K | 55 y, M |  |  |
|  | 199 | Beijing | | c.770 A>A/G;  c.1395 T>G/T p.T | p.Tyr>Cys | median onset age of 30.0 (23.0, 37.0) years  Males 19  Female 6 | Zhao 2018 [29] |  |
|  | 200 |  |  | c.691T >A/T;  c.770A>A/G | p.Phe231Ile  p. 257 Tyr>Cys |  |  |  |
|  | 201 |  |  | c.872T>T/G  c.1227A>A/C | p.Val29Gly  p.Leu409Phe |  |  |  |
|  | 202 |  |  | c.770 A>A/G  c.1211 T>C/T | p.Tyr>Cys p.Met>Thr |  |  |  |
|  | 203 |  |  | c.1123C>A  c.617A>T | p.P375T  p.H206L |  |  |  |
|  | 204 |  |  | c.3G>C  c.152G>A | p.M1I  p.R51Q |  |  |  |
|  | 205 |  |  | c.617A>T  c.872T>T/G | p.H206L p.Val29Gly |  |  |  |
|  | 206 |  |  | c.389A>A/T;  c.1597C>C/T | p.Asp130Val p.Gln533Term |  |  |  |
|  | 207 |  |  | c.691T>A/T;  c.1211 T>C/T | p.Phe231Ile p.Met>Thr |  |  |  |
|  | 208 |  |  | c.535A>G  c.1044A>C | p.K179E  p.L348F |  |  |  |
|  | 209 |  |  | c.242T>C;  c.295C>T | p.L81P  p.R99C |  |  |  |
|  | 210 |  |  | c.1028T>C;  c.1044A>C | p.M343T  p.L348F |  |  |  |
|  | 211 |  |  | c.1227A>C  c.1395T>G | p. L409F;  p.Y465* |  |  |  |
|  | 212 |  |  | c.872T>T/G;  c.1211T>T/C | p.Val291Gly p.Met404Thr |  |  |  |
|  | 213 |  |  | c.1227A>C;  Exon10 c.1281_1282del | p.L409 F  p.T427fs |  |  |  |
|  | 214 |  |  | c.53C>G  c.587A>G | p.A18G;  p.Y196C |  |  |  |
|  | 215 |  |  | c.61T>C  c.1271C>G | p.S21P;  p.T424S |  |  |  |
|  | 216 |  |  | c.315G>A  c.315G>A | p.M105I  p.M105I |  |  |  |
|  | 217 |  |  | c.250G>A | p.Ala84Thr |  |  |  |
|  | 218 |  |  | c.1395 T>G/T | p.Tyr>Term |  |  |  |
|  | 219 |  |  | c.1227A>C | p.Leu409Phe |  |  |  |
|  | 220 |  |  | c.1227A>A/C | p.Leu409Phe |  |  |  |
|  | 221 |  |  | c.1454C>C/G | p.Thr485Ser |  |  |  |
|  | 222 |  |  | c.176-2A>G | p.I72T |  |  |  |
|  | 223 |  |  | c.92C>T | p.Thr31Ile |  |  |  |
|  | 1. 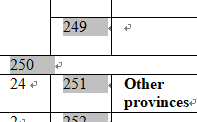 | Beijing | | c.770A>G  c.1450T>C |  | 41 y, M | Wang 2016 [30] |  |
|  |  |  |  | c.65A>G;  c.242T>C |  | 43 y, M |  |  |
|  |  |  |  | c.65A>G ;  c.242T>C |  | 42 y, M |  |  |
|  |  |  |  | c.242T>C ;  c.295C>T |  | 32 y, F |  |  |
|  |  |  |  | c.1227A>C |  | 36 y, M |  |  |
|  |  |  |  | c.770A>G;  c.920C>G |  | 54 y, M |  |  |
|  |  | Beijing | | c.770A>G  - | p.Y257C  - | Median onset age of 27.9±9 (12, 49) years； | Wang 2011 [31] |  |
|  |  | Beijing | | c.IVS6 +1G>A  - | -  - |  |  |  |
|  | 232-233 | Beijing  2 cases | | c.3G >C  c.770A>G | p.M1I  p.Y257C |  |  |  |
|  | 234-235 | Beijing  2 cases | | c.IVS6 +1G>A  c.1227A > C | -  p.L409F |  |  |  |
|  | 236-237 | Beijing  2 cases | | c.1227A > C  c.389A>T | p.L409F  p.D130V |  |  |  |
|  | 238 |  | | c.770A > G  c.993T>G | p.Y257C  p.N331K |  |  |  |
|  | 239 |  | | c.250 G>A  c.1227A > C | p.A84T  p.L409F |  |  |  |
|  |  |  | | c.998A > G  c.1450T>C | p.Y333C  p.W484R |  |  |  |
|  |  |  | | c.770A > G  c.IVS7+2T>C | p.Y257C  - |  |  |  |
|  |  |  | | c. 1703T > G  - | p.F568S  - |  |  |  |
|  |  |  | | c.770A > G  - | p.Y257C  - |  |  |  |
|  |  |  | | c.1227A > C  c.1717 C > T | p.L409F  p.Q573Stop |  |  |  |
|  |  |  | | c.821 G>A  c.1227A > C | p.G274E  p.L409F |  |  |  |
|  |  |  | | c.389 A > T  c.643G>A | p.D130V  p.A215T |  |  |  |
|  |  |  | | c.1675C > T  c.1227A > C | p.R559 stop  p.L409F |  |  |  |
|  |  |  | | c.251 C > T  - | p.A84V  - |  |  |  |
|  |  |  | | c.770A > G  c.770A > G | p.Y257C  p.Y257C |  |  |  |
|  |  |  | | c.770A > G  c.389A> T | p.Y257C  p.D130V |  |  |  |
|  |  |  | | c.770A > G  c.1763A> T | p.Y257C  H588L |  |  |  |
|  |  |  | | c.770A > G  c.1450T> C | p.Y257C  p.W484R |  |  |  |
|  | | | | | | | |  |
| 24 |  | | China, Other provinces |  |  |  |  |  |
| 2 |  | | Guangxi | c.524G>A  c.1450T>C | p.R175H  p.W484R | 27 y, M | Fan 2018[32] |  |
|  |  | |  | c.1157G>A  c.1450T>C | p.G286D  p.W484R | 30 y, M |  |  |
| 2 |  | | Wuhan | c.1331T>C  c.824C>T | p.Val444Ala | 12 y, F | Cheng 2017 [33] |  |
|  |  | |  | c.177insT  c.1474T>C | p.D59fs | 6 y, M |  |  |
| 13 |  | | Jiangxi | c.1395T4G | p.Y465X | 48y, F | Zhu 2014 [34]  Li 2015 [35] |  |
|  |  | |  | c. 1773-1774delAT | p.T591TfsX2 | 39 y, M |  |  |
|  |  | |  | c.1084G4A | p.G362R | 31 y, M |  |  |
|  |  | |  | c.3G4C | p.M1I | 33 y, F |  |  |
|  |  | |  | 1c.1399G4A | p.G467R | 38 y, M |  |  |
|  |  | |  | c.389A4T  c.1227A4C | p.D130V  p.L409F | 35 y, F |  |  |
|  |  | |  | c.303T4A  c.1810G4T | p.C101Xn  p.V604Ln | 57 y, M |  |  |
|  |  | |  | c.518T4G  c.1211T4C | p.I173Mn  p.M404T | 36 y, M |  |  |
|  |  | |  | c.295C4T  c.1586A4G | p.R99Cn  p.H529Rn | 35 y, M |  |  |
|  |  | |  | c.715G4A  c.1810G4T | p.A239T  p.V604Ln | 32 y, M |  |  |
|  |  | |  | c.295C4T  c.821G4A | p.R99Cn  p.G274E | 26 y, M |  |  |
|  |  | |  | c.524G4A  c.1828G4A | p.R175L  p.G610R | 56 y, F |  |  |
|  |  | |  | c.770A4G  c.1395T4G  c. 1773-1774delAT | p.Y257C  p.Y465X | 39 y, M |  |  |
| 1 |  | | Jiangxi | c.250G>A  c389A>T | p.A84T  p.D130V | 41y, M | Li 2015 [35] |  |
| 1 |  | | Zhejiang,  Hangzhou | c.1601C＞T  c. 1787A＞G | p．Pro534Leu  p．Asp596Gly | 8y, M | Tong 2017 [36] |  |
| 1 |  | | Hunan | c.1773_1774 del A  c.389A＞T | p.(Cys592※) | 7 y, M | Tang 2017 [37] |  |
| 1 |  | | Nanchang | C.770A>G  920C>G | p.T257C  p.S307C | 46 y, M | Peng 2015[38] |  |
| 3 | 274-276 | | Jilin | c.770A>G  c.1669G>A | Tyr257Cys  Glu557Lys | n.a | Zhao 2014 [39] |  |
|  | | | | | | | |  |
| 16 |  | | Japan | c.1367C>T  c.1367C>T | p.P456L | 58 y, M | Yamada, 2016 [40] |  |
|  |  | | Japan | c.890G>T  c.950C>G | p.W297L  p.P317R | 31 y, M |  |  |
|  |  | | Japan | 890G > T/W297L  950C > G/P317R) |  | 31 y, M | Shioya, 2014 [41] |  |
|  |  | | Japan | c.1211T>C  c.1786G>A | p.M404T  p.D596N | 46y, M | Izumi 2011 [42] |  |
|  |  | |  | c.1531G>A  c.1809G>A | p.D511N  p.W603* | 36y, M | Sugai 2012 [43] |  |
|  |  | |  | c.922T>G  c.? | p.F308V  p.? | 5m, M | Yotsumoto 2008 [44] |  |
|  |  | |  | c.1208C>T  c.1208C>T | p.A403V  p.A403V | 6m, M |  |  |
|  |  | |  | c.1084G>A  c.1601C>T | p.G362R  p.P534L | 8m, M |  |  |
|  |  | |  | c.1096C>T  c.1675C>T | p.L366F  p.R559* | 16m, F |  |  |
|  |  | |  | c.1096C>T  c.? | p.L366F  p.? | 22m, F |  |  |
|  |  | |  | c.524G>A  c.1774T>C | p.R175H  p.C592R | 5y, F |  |  |
|  |  | |  | c.1367C>T  c.1367C>T | p.P456L  p.P456L | 40y, M |  |  |
|  |  | |  | c.524G>A  c.1774T>G | p.R175H  p.C592R | Childhood ,F | Ishii 2010 [45] |  |
|  |  | |  | c.1519T>G | p.Y507D | n.a,F | Ohkuma 2009 [46] |  |
|  |  | |  | c.1208C>T  c.1208C>T | p.A403V  p.A403V | n.a,M |  |  |
|  |  | |  | c.524G>A  c.1774T>G | p.R175H  p.C592R | n.a,F |  |  |
|  | | | | | | | |  |
| 2 |  | | **Korea** | c.1354A>G | p.Arg452Gly | 12 d, M | Kim 2018[47] |  |
|  |  | |  | c.831+3A>C |  | 7 d, M |  |  |
|  | | | | | | | |  |
| 87 |  | | **Other Countries** |  |  |  |  |  |
| 3 |  | | South Africa | c.1067G > A +/+; |  | 1 week, M | Van Der Westhuizen 2018 [48] |  |
|  |  | |  | c.1067G > A +/−;  c.1448C > T +/−; | p.Gly356Glu p.Pro483Leu  (377656387) | 2y, F |  |  |
|  |  | |  | c.1067G > A +/−;  c.1448C > T +/−; | p.Gly356Glu  p.Pro483Leu (377656387) | 4 y, F |  |  |
| 5 |  | | Italy | c.412C>T, exon 4;  c.1531G>A ,exon 12 | p.L138F  p.D511N, | 35 y，F | Angelini, 2017 [49] |  |
|  |  | |  | c.560C>T; exon 5  c.1027T>C, exon 11 | p.A187V, p.W343R, | 38 y, F |  |  |
|  |  | |  | c.451A>G, exon 4  c.1649T>G, exon 12 | p.T151A  p.L550P | 23 y, F |  |  |
|  |  | |  | c.152G>A, exon 2 | p.R51Q | 16 y, F |  |  |
|  |  | |  | c.606+5insT, exon 5 |  | 33 y, M |  |  |
| 5 |  | | USA | c.245>T  c.524G>A | p.Ser82Phe  p.Arg175His | 33 y，F | Whitaker, 2015[50] |  |
|  |  | | USA | c.820G>T  c.1601C>T | p.G274*  p.P534L | 4 m,M | Wolfe 2010[51] |  |
|  |  | | USA | c.79C>T  c.79C>T | p.P27S  p.P27S | 2.5 y，F | Pollard 2010 [52] |  |
|  |  | |  | c.79C>T  c.79C>T | p.P27S  p.P27S | 8 y, M |  |  |
|  |  | | USA | c.625G>A  c.1852T>C | p.D218N  p.*618Q (original stopcodon lost, results in prolonged protein) | 3.5 m ,M | Angle 2008[53] |  |
|  |  | |  | c.731T>C  c.814G>A | p.F244S  p.G272R | 12m ,M |  |  |
| 1 |  | | France | c.877C>G  c.? | p.H293D  p.? | 55 y, F | Kaminsky 2011 |  |
| 13 |  | | France Chinese origin | c.250G>A [14] FAD binding c.524G>A [13] | p.Ala84Thr | 18 y, F | Behin 2015 [54]  Maillart 2010 |  |
|  |  | | France | c.571G>A FAD binding  c.1331T>C | p.Gly191Ser  p.Val444Ala | 15y, M | Behin 2015 [54]  Maillart 2010[55] |  |
|  |  | |  | c.622G>C Linker  c.1241_1246del FAD binding | p.Asp208His p.Ile414_Phe415del | 32y, F |  |  |
|  |  | |  | c.877C>G UQ binding [3]  c.1691-3C>G [17] | p.His293Asp  splicing defect | 18y, M |  |  |
|  |  | |  | c.1732C>T  ? | p.Arg578Trp  - | 18y, F |  |  |
|  |  | |  | c.79C>T  c.1471_1473delins8 | p.Pro27Ser p.Ser491GlnfsX3 | 39y, M |  |  |
|  |  | |  | c.406-2A>G  c.728T>C [11] | Splicing defect  p.Ile243Thr | 35y, F |  |  |
|  |  | |  | c.1366C>T UQ binding  c.1366C>T | p.Pro456Ser p.Pro456Ser | 22y, F |  |  |
|  |  | |  | c.1366C>T UQ binding  c.1366C>T | p.Pro456Ser p.Pro456Ser | 35y, F |  |  |
|  |  | |  | c.877C>G [3] UQ binding  ? | p.His293Asp  - | 55y, F |  |  |
|  |  | |  | c.245C>T [31] FAD binding c.245C>T [31] | p.Ser82Phe p.Ser82Phe | 14y, M |  |  |
|  |  | |  | c.1366C>T UQ binding  c.1366C>T | p.Pro456Ser p.Pro456Ser | 25y, M |  |  |
|  |  | |  | c.769T>C FAD binding  c.769T>C | p.Tyr257His p.Tyr257His | 24y, F |  |  |
| 1 |  | | Finland | c.1141G>C  homozygous | p.Gly381Arg | Infant, m | Vieira 2017 [56] |  |
| 23 |  | | Turkey | c.1130T>C | p.Leu377pro | 21y，F | Yıldız 2019 [57] |  |
|  |  | |  | c.1448C>T | p.Pro483Leu | 12 y，F |  |  |
|  |  | |  | c.1448C>T | p.Pro483Leu | 10 y，F |  |  |
|  |  | |  | c.1130T>C | p.Leu377pro | 5 y，F |  |  |
|  |  | |  | c.1130T>C | p.Leu377pro | F |  |  |
|  |  | |  | c.1130T>C | p.Leu377pro | 12 y，F |  |  |
|  |  | |  | c.1130T>C | p.Leu377pro | 16 y，F |  |  |
|  |  | |  | c.1130T>C | p.Leu377pro | 16 y,M |  |  |
|  |  | |  | c.1130T>C | p.Leu377pro | 9 y，F |  |  |
|  |  | |  | c.1130T>C | p.Leu377pro | 7 y，F |  |  |
|  |  | |  | c.1130T>C | p.Leu377pro | F |  |  |
|  |  | |  | c.1130T>C | p.Leu377pro | 2 y，F |  |  |
|  |  | |  | c.1130T>C | p.Leu377pro | 1y, M |  |  |
|  |  | |  | c.1448C>T | p.Pro483Leu | 4m, F |  |  |
|  |  | |  | c.1448C>T | p.Pro483Leu | 4m ,M |  |  |
|  |  | |  | c.1130T>C | p.Leu377pro | 13 y, M |  |  |
|  |  | |  | c.1130T>C | p.Leu377pro | 10 y, M |  |  |
|  |  | |  | c.1130T>C | p.Leu377pro | 10 y, M |  |  |
|  |  | |  | c.1790C>T | p.Pro597 L eu | 14 y，F |  |  |
|  |  | |  | c.1141G>C | p.Gly381Arg | 1d, M |  |  |
|  |  | |  | c.1165C>A | p.Pro389Thr | 50 d, ,M |  |  |
|  |  | |  | c.1141G>C  c.1198_1201delACTC | p.Gly381Arg  p.His401Glnfs83 | 1 d ,M |  |  |
|  |  | |  | c.1524delA  c.1524delA |  | 1d,M |  |  |
| 2 |  | | Turkey (report in Germany) | c.1117-2A > G  c.1117-2A > G |  | infant | Hacki, 2017 [58] |  |
|  |  | | Turkey | c.1384C>G  c. 1468+ldelG | p.L462V | 20 y, M | Gorukmez 2015[59] |  |
| 1 |  | | Ireland | c.51_52ins  c.1367>T | p.A18Cfs*5  p.P456L | 6 mo, F | Fitzgerald 2013 [60] |  |
| 11 |  | | Germany | c.1130T>C  c.1130T>C | p.L377P  p.L377P | 22 y, M | Scheicht 2013[61] |  |
|  |  | | Germany | c.1544G>T  c.1544G>T | p.S515I  p.S515I | 25 y，F | Rosenbohm 2014[62] |  |
|  |  | | Germany | c.1218 G>C  c.1698 C>T | p.G296R  p.P456P | 41 y, M | Lämmer 2011[63] |  |
|  |  | | Germany | c.728T>C  c.881C>T | p.I243T  p.T294I | 42 y, M | Köppel 2006[64] |  |
|  |  | | Germany | c.1448C>T  c.1448C>T | p.P483L  p.P483L | 12 y，F | Gempel 2007[65] |  |
|  |  | |  | c.1130T>C  c.1130T>C | p.L377P  p.L377P | 13 y, M |  |  |
|  |  | |  | c.1130T>C  c.1130T>C | p.L377P  p.L377P | 14 y，F |  |  |
|  |  | |  | c.1130T>C  c.1130T>C | p.L377P  p.L377P | 12 y，F |  |  |
|  |  | |  | c.1130T>C  c.1130T>C | p.L377P  p.L377P | F |  |  |
|  |  | | Germany | c.1367C>T  c.1768A>G | p.P456L  p.K590E | 32 y，F | Gempel 2007[25]  Horvath 2006[66] |  |
|  |  | |  | c.1130T>C  c.1130T>C | p.L377P  p.L377P | 29 y, M |  |  |
| 3 |  | | Canada | c.1601C>T  c.1601C>T | p.P534L  p.P534L | 4 y，F | Trakadis 2012 [67] |  |
|  |  | |  | c.1601C>T  c.1601C>T | p.P534L  p.P534L | 21 y，F |  |  |
|  |  | |  | c.1601C>T  c.1601C>T | p.P534L  p.P534L | M |  |  |
| 18 |  | | Demark | c.413T>G  c.IVS3+3A>T | p.L138R  truncated transcript | 6m,F | Olsen 2004 [68]  Turnbull 1988 [69] |  |
|  |  | |  | c.806A>T  c.1448C>T | p.Q269L  p.P483L | 2 y, M | Olsen 2007 [70]  Gregersen 1982[71] Gregersen 1986[72] |  |
|  |  | |  | c.806A>T  c.1448C>T | p.Q269L  p.P483L | 2 y, M |  |  |
|  |  | |  | c.244T>C  c.244T>C | p.S82P  p.S82P | 3 y, M | Olsen 2007 [70]  Ramos 1995 |  |
|  |  | |  | c.1367C>T  c.1367C>T | p.P456L  p.P456L | 14 y，F | Olsen 2007 [70]  Henderson 2002 |  |
|  |  | |  | c.1367C>T  c.1367C>T | p.P456L  p.P456L | F | Olsen 2007 [70] |  |
|  |  | |  | c.1351G>C  c.1351G>C | p.V451L  p.V451L | 9 y，F |  |  |
|  |  | |  | c.1351G>C  c.1351G>C | p.V451L  p.V451L | 8 y，F |  |  |
|  |  | |  | c.334C>T  c.1366C>A | p.H112Y  p.P456T | 13 y，F | Olsen 2007 [70]  Beresford 2006[69] |  |
|  |  | |  | c.1001T>C  c.1367C>T | p.L334P  p.P456L | 16 y，F | Olsen 2007 [70] |  |
|  |  | |  | c.1001T>C  c.1367C>T | p.L334P  p.P456L | -,F |  |  |
|  |  | |  | c.1445A>T  c.? | p.E482V  p.? | 21 y，F | Olsen 2007 [70]  Moore 1998 |  |
|  |  | |  | c.51_52insT  c.508G>T | p.A18Cfs*5  p.G170C | 22 y，F | Olsen 2007 [70] |  |
|  |  | |  | c.51_52insT  c.1367C>T | p.A18Cfs*5  p.P456L | 3 y，F |  |  |
|  |  | |  | c.?  c.1285G>C | p.A12fs  p.G429R | 33 y, M |  |  |
|  |  | |  | c.1060G>T  c.1351G>C | p.354*  P.v451L | 31 y，F | Olsen 2007 [70]  Stojanovic 2000 [73] |  |
|  |  | |  | c.1074G*>*C  c.1074G*>*C | p.R358S  p.R358S | -,M | Olsen 2005 [74] |  |
|  |  | |  | c.1074G*>*C  c.1074G*>*C | p.R358S  p.R358S | -,F |  |  |

1. Xu HL, Lian YJ, Chen X, Zhang L, Cheng X. Two novel ETFDH mutations in a patient with lipid storage myopathy. Chin Med J (Engl). 2019; doi: 10.1097/CM9.0000000000000310.

2. Chen Y LH, Yuan X, Wang X, Teng J. Clinical analysis of riboflavin-reactive lipid storage myopathy in two families. Chinese Journal of Practical Nervous Diseases. 2018;21 24:2692-6.

3. Cui YJ, Song CL, Cheng YB. [Paroxysmal muscle weakness, liver enlargement, and hypoglycemia in a boy]. Zhongguo Dang Dai Er Ke Za Zhi. 2017;19 10:1104-8.

4. Chen M, Peng J, Wei W, Wang R, Xu H, Liu H. A novel ETFDH mutation in an adult patient with late-onset riboflavin-responsive multiple acyl-CoA dehydrogenase deficiency. Int J Neurosci. 2018;128 3:291-4; doi: 10.1080/00207454.2017.1380641.

5. Zhen C, Li Y, Tian F. Diagnosis and management of a twin brother and sister with Glutaric Aciduria Type II. Journal of Zhengzhou University(Medical Sciences). 2019;54 2:309-12.

6. Zhuo Z, Jin P, Li F, Li H, Chen X, Wang H. A case of late-onset riboflavin responsive multiple acyl-CoA dehydrogenase deficiency (MADD) with a novel mutation in ETFDH gene. J Neurol Sci. 2015;353 1-2:84-6; doi: 10.1016/j.jns.2015.04.011.

7. Fu HX, Liu XY, Wang ZQ, Jin M, Wang DN, He JJ, et al. Significant clinical heterogeneity with similar ETFDH genotype in three Chinese patients with late-onset multiple acyl-CoA dehydrogenase deficiency. Neurol Sci. 2016;37 7:1099-105; doi: 10.1007/s10072-016-2549-2.

8. Chen HZ, Ming J, Cai NQ, Lin XD, Liu XY, Xu LQ, et al. Rhabdomyolysis and respiratory insufficiency due to the common ETFDH mutation of c.250G>A in two patients with late-onset multiple acyl-CoA dehydrogenase deficiency. Chin Med J (Engl). 2019; doi: 10.1097/CM9.0000000000000288.

9. Wang ZQ, Chen XJ, Murong SX, Wang N, Wu ZY. Molecular analysis of 51 unrelated pedigrees with late-onset multiple acyl-CoA dehydrogenation deficiency (MADD) in southern China confirmed the most common ETFDH mutation and high carrier frequency of c.250G>A. J Mol Med (Berl). 2011;89 6:569-76; doi: 10.1007/s00109-011-0725-7.

10. Er TK, Liang WC, Chang JG, Jong YJ. High resolution melting analysis facilitates mutation screening of ETFDH gene: applications in riboflavin-responsive multiple acyl-CoA dehydrogenase deficiency. Clin Chim Acta. 2010;411 9-10:690-9; doi: 10.1016/j.cca.2010.01.033.

11. Chien YH, Lee NC, Chao MC, Chen LC, Chen LH, Chien CC, et al. Fatty Acid oxidation disorders in a chinese population in taiwan. JIMD Rep. 2013;11:165-72; doi: 10.1007/8904_2013_236.

12. Lan MY, Fu MH, Liu YF, Huang CC, Chang YY, Liu JS, et al. High frequency of ETFDH c.250G>A mutation in Taiwanese patients with late-onset lipid storage myopathy. Clin Genet. 2010;78 6:565-9; doi: 10.1111/j.1399-0004.2010.01421.x.

13. Liang WC, Ohkuma A, Hayashi YK, Lopez LC, Hirano M, Nonaka I, et al. ETFDH mutations, CoQ10 levels, and respiratory chain activities in patients with riboflavin-responsive multiple acyl-CoA dehydrogenase deficiency. Neuromuscul Disord. 2009;19 3:212-6; doi: 10.1016/j.nmd.2009.01.008.

14. Lu J, Ji L. [Mutation analysis for a family affected with riboflavin responsive-multiple acyl-CoA dehydrogenase deficiency]. Zhonghua Yi Xue Yi Chuan Xue Za Zhi. 2014;31 4:428-32; doi: 10.3760/cma.j.issn.1003-9406.2014.04.004.

15. Goh LL, Lee Y, Tan ES, Lim JSC, Lim CW, Dalan R. Patient with multiple acyl-CoA dehydrogenase deficiency disease and ETFDH mutations benefits from riboflavin therapy: a case report. BMC Med Genomics. 2018;11 1:37; doi: 10.1186/s12920-018-0356-8.

16. Cao JZ, C; Li, Y; Yang, J; Liang, Y; Feng, S; Zhang, X; Li, J; Zhang, Y. Clinical characteristics and gene mutation analysis of riboflavin-responsive lipid storage myopathy: report of 3 cases in 2 families and review of literature. Chinese Journal of Contemporary Neurology and Neurosurgery. 2014;14 6:479-84; doi: 10.3969/j.issn.1672-6731.2014.06.004.

17. Kong LE, Zhuang SZ, Li CM, Chen LK, Zhang TT, Qiu JH, et al. Study of ETFDH gene mutations in a family with riboflavin-responsive lipid storage myopathy. Journal of Apoplexy and Nervous Diseases 2018;35 10:927-9.

18. Dai D, Wen F, Zhou S, Chen S. [Clinical features and gene mutations in a patient with multiple aeyl-CoA dehydrogenase deficiency with severe fatty liver]. Zhonghua Yi Xue Yi Chuan Xue Za Zhi. 2016;33 2:191-4; doi: 10.3760/cma.j.issn.1003-9406.2016.02.014.

19. Law LK, Tang NL, Hui J, Fung SL, Ruiter J, Wanders RJ, et al. Novel mutations in ETFDH gene in Chinese patients with riboflavin-responsive multiple acyl-CoA dehydrogenase deficiency. Clin Chim Acta. 2009;404 2:95-9; doi: 10.1016/j.cca.2009.02.015.

20. Santananukarn M, Amornvit J, Pasutharnchat N, Jongpiputvanich S. Needle EMG, a jigsaw to disclose lipid storage myopathy due to Multiple Acyl-CoA Dehydrogenase Deficiency, a case report. Am J Phys Med Rehabil. 2019; doi: 10.1097/PHM.0000000000001230.

21. Wasant P, Kuptanon C, Vattanavicharn N, Liammongkolkul S, Ratanarak P, Sangruchi T, et al. Glutaric aciduria type 2, late onset type in Thai siblings with myopathy. Pediatr Neurol. 2010;43 4:279-82; doi: 10.1016/j.pediatrneurol.2010.05.018.

22. Xue Y, Zhou Y, Zhang K, Li L, Kayoumu A, Chen L, et al. Compound heterozygous mutations in electron transfer flavoprotein dehydrogenase identified in a young Chinese woman with late-onset glutaric aciduria type II. Lipids Health Dis. 2017;16 1:185; doi: 10.1186/s12944-017-0576-5.

23. Xi J, Wen B, Lin J, Zhu W, Luo S, Zhao C, et al. Clinical features and ETFDH mutation spectrum in a cohort of 90 Chinese patients with late-onset multiple acyl-CoA dehydrogenase deficiency. J Inherit Metab Dis. 2014;37 3:399-404; doi: 10.1007/s10545-013-9671-6.

24. Wen B, Li D, Li W, Zhao Y, Yan C. Multiple acyl-CoA dehydrogenation deficiency as decreased acyl-carnitine profile in serum. Neurol Sci. 2015;36 6:853-9; doi: 10.1007/s10072-015-2197-y.

25. . !!! INVALID CITATION !!!

26. Zhao ZN, Bao MX, Ma GT, Liu XM, Xu WJ, Sun ZW, et al. A case of late-onset riboflavin responsive multiple acyl-CoA dehydrogenase deficiency with novel mutations in ETFDH gene. CNS Neurosci Ther. 2012;18 11:952-4; doi: 10.1111/cns.12007.

27. Hu X, Li L, Mi R, Zhao Y, Li C. A family affected by multiple acyl-CoA dehydrogenase deficiency due to compound heterozygous mutations on ETFDH and literature review. Chinese Journal of Neonatology. 2018;33 3:205-9; doi: 10.3760/cma.j.issn.2096-2932.2018.03.012.

28. Hong D, Yu Y, Wang Y, Xu Y, Zhang J. Acute-onset multiple acyl-CoA dehydrogenase deficiency mimicking Guillain-Barre syndrome: two cases report. BMC Neurol. 2018;18 1:219; doi: 10.1186/s12883-018-1221-2.

29. Zhao YW, Liu XJ, Zhang W, Wang ZX, Yuan Y. Muscle Magnetic Resonance Imaging for the Differentiation of Multiple Acyl-CoA Dehydrogenase Deficiency and Immune-mediated Necrotizing Myopathy. Chin Med J (Engl). 2018;131 2:144-50; doi: 10.4103/0366-6999.222323.

30. Wang Z, Hong D, Zhang W, Li W, Shi X, Zhao D, et al. Severe sensory neuropathy in patients with adult-onset multiple acyl-CoA dehydrogenase deficiency. Neuromuscul Disord. 2016;26 2:170-5; doi: 10.1016/j.nmd.2015.12.002.

31. Wang Y, Zhao DH, Hong DJ, Wang CX, Y. Y. Hot spot mutations in electron transfer flavoprotein dehydrogenase gene of riboflavin responsive lipid storage myopathy in 20 Chinese families. Chinese Journal of Neurology. 2011;44 5:309-13; doi: 10.3760/cma.j.issn.1006-7876.2011.05.005.

32. Fan X, Xie B, Zou J, Luo J, Qin Z, D'Gama AM, et al. Novel ETFDH mutations in four cases of riboflavin responsive multiple acyl-CoA dehydrogenase deficiency. Mol Genet Metab Rep. 2018;16:15-9; doi: 10.1016/j.ymgmr.2018.05.007.

33. Cheng YY, Tang Y, Liu AJ, Wei L, Lin L, Zhang J, et al. [Clinical features and ETFDH mutations of children with late-onset glutaric aciduria type II: a report of two cases]. Zhongguo Dang Dai Er Ke Za Zhi. 2017;19 9:975-8.

34. Zhu M, Zhu X, Qi X, Weijiang D, Yu Y, Wan H, et al. Riboflavin-responsive multiple Acyl-CoA dehydrogenation deficiency in 13 cases, and a literature review in mainland Chinese patients. J Hum Genet. 2014;59 5:256-61; doi: 10.1038/jhg.2014.10.

35. Li XM. Clinical characteristic of lipid deposit disease in Jiangxi area and ETFA, ETFB, ETFDH gene mutation analysis. Nanchan University 2015.

36. Tong F, Yan R, Zhao Z. One case report of late onset riboflavin-responsive multiple acylCoA dehydrogenase deficiency,. Chinese Journal of Child Health Care. 2017;25 11:1186-8.

37. Tang Y, Ouyang W, Jiang T, Tan L, Su Y. Clinical manifestation and gene analysis of multiple acyl-coa dehydrogenase deficiency with transaminase increase. Chinese Pediatrics Of Integrated Traditional And Western Medicine. 2017;9 5:456-8.

38. Peng Y, Zhu M, Zheng J, Zhu Y, Li X, Wei C, et al. Bent spine syndrome as an initial manifestation of late-onset multiple acyl-CoA dehydrogenase deficiency: a case report and literature review. BMC Neurol. 2015;15:114; doi: 10.1186/s12883-015-0380-7.

39. Zhao F. Clinical, pathology, biochemical and genetic analysis of one family with lipid diposit myopathy Jilin University Degree thesis. 2014.

40. Yamada K, Kobayashi H, Bo R, Takahashi T, Purevsuren J, Hasegawa Y, et al. Clinical, biochemical and molecular investigation of adult-onset glutaric acidemia type II: Characteristics in comparison with pediatric cases. Brain Dev. 2016;38 3:293-301; doi: 10.1016/j.braindev.2015.08.011.

41. Shioya A, Takuma H, Yamaguchi S, Ishii A, Hiroki M, Fukuda T, et al. Amelioration of acylcarnitine profile using bezafibrate and riboflavin in a case of adult-onset glutaric acidemia type 2 with novel mutations of the electron transfer flavoprotein dehydrogenase (ETFDH) gene. J Neurol Sci. 2014;346 1-2:350-2; doi: 10.1016/j.jns.2014.08.040.

42. Izumi R, Suzuki N, Nagata M, Hasegawa T, Abe Y, Saito Y, et al. A case of late onset riboflavin-responsive multiple acyl-CoA dehydrogenase deficiency manifesting as recurrent rhabdomyolysis and acute renal failure. Intern Med. 2011;50 21:2663-8.

43. Sugai F, Baba K, Toyooka K, Liang WC, Nishino I, Yamadera M, et al. Adult-onset multiple acyl CoA dehydrogenation deficiency associated with an abnormal isoenzyme pattern of serum lactate dehydrogenase. Neuromuscul Disord. 2012;22 2:159-61; doi: 10.1016/j.nmd.2011.08.004.

44. Yotsumoto Y, Hasegawa Y, Fukuda S, Kobayashi H, Endo M, Fukao T, et al. Clinical and molecular investigations of Japanese cases of glutaric acidemia type 2. Mol Genet Metab. 2008;94 1:61-7; doi: 10.1016/j.ymgme.2008.01.002.

45. Ishii K, Komaki H, Ohkuma A, Nishino I, Nonaka I, Sasaki M. Central nervous system and muscle involvement in an adolescent patient with riboflavin-responsive multiple acyl-CoA dehydrogenase deficiency. Brain Dev. 2010;32 8:669-72; doi: 10.1016/j.braindev.2009.08.008.

46. Ohkuma A, Noguchi S, Sugie H, Malicdan MC, Fukuda T, Shimazu K, et al. Clinical and genetic analysis of lipid storage myopathies. Muscle Nerve. 2009;39 3:333-42; doi: 10.1002/mus.21167.

47. Kim YJ, Ko JM, Song J, Lee KA. Clinical Features of Multiple Acyl-CoA Dehydrogenase Deficiency With ETFDH Variants in the First Korean Cases. Ann Lab Med. 2018;38 6:616-8; doi: 10.3343/alm.2018.38.6.616.

48. van der Westhuizen FH, Smuts I, Honey E, Louw R, Schoonen M, Jonck LM, et al. A novel mutation in ETFDH manifesting as severe neonatal-onset multiple acyl-CoA dehydrogenase deficiency. J Neurol Sci. 2018;384:121-5; doi: 10.1016/j.jns.2017.11.012.

49. Angelini C, Tavian D, Missaglia S. Heterogeneous Phenotypes in Lipid Storage Myopathy Due to ETFDH Gene Mutations. JIMD Rep. 2017; doi: 10.1007/8904_2017_27.

50. Whitaker CH, Felice KJ, Silvers D, Wu Q. Fulminant lipid storage myopathy due to multiple acyl-coenzyme a dehydrogenase deficiency. Muscle Nerve. 2015;52 2:289-93; doi: 10.1002/mus.24552.

51. Wolfe LA, He M, Vockley J, Payne N, Rhead W, Hoppel C, et al. Novel ETF dehydrogenase mutations in a patient with mild glutaric aciduria type II and complex II-III deficiency in liver and muscle. J Inherit Metab Dis. 2010;33 Suppl 3:S481-7; doi: 10.1007/s10545-010-9246-8.

52. Pollard LM, Williams NR, Espinoza L, Wood TC, Spector EB, Schroer RJ, et al. Diagnosis, treatment, and long-term outcomes of late-onset (type III) multiple acyl-CoA dehydrogenase deficiency. J Child Neurol. 2010;25 8:954-60; doi: 10.1177/0883073809351984.

53. Angle B, Burton BK. Risk of sudden death and acute life-threatening events in patients with glutaric acidemia type II. Mol Genet Metab. 2008;93 1:36-9; doi: 10.1016/j.ymgme.2007.09.015.

54. Behin A, Acquaviva-Bourdain C, Souvannanorath S, Streichenberger N, Attarian S, Bassez G, et al. Multiple acyl-CoA dehydrogenase deficiency (MADD) as a cause of late-onset treatable metabolic disease. Rev Neurol (Paris). 2016;172 3:231-41; doi: 10.1016/j.neurol.2015.11.008.

55. Maillart E, Acquaviva-Bourdain C, Rigal O, Brivet M, Jardel C, Lombes A, et al. [Multiple acyl-CoA dehydrogenase deficiency (MADD): a curable cause of genetic muscular lipidosis]. Rev Neurol (Paris). 2010;166 3:289-94; doi: 10.1016/j.neurol.2009.05.009.

56. Vieira P, Myllynen P, Perhomaa M, Tuominen H, Keski-Filppula R, Rytky S, et al. Riboflavin-Responsive Multiple Acyl-CoA Dehydrogenase Deficiency Associated with Hepatoencephalomyopathy and White Matter Signal Abnormalities on Brain MRI. Neuropediatrics. 2017;48 3:194-8; doi: 10.1055/s-0037-1601447.

57. Yildiz Y, Talim B, Haliloglu G, Topaloglu H, Akcoren Z, Dursun A, et al. Determinants of Riboflavin Responsiveness in Multiple Acyl-CoA Dehydrogenase Deficiency. Pediatr Neurol. 2019; doi: 10.1016/j.pediatrneurol.2019.06.015.

58. Hackl A, Mehler K, Gottschalk I, Vierzig A, Eydam M, Hauke J, et al. Disorders of fatty acid oxidation and autosomal recessive polycystic kidney disease-different clinical entities and comparable perinatal renal abnormalities. Pediatr Nephrol. 2017;32 5:791-800; doi: 10.1007/s00467-016-3556-5.

59. Gorukmez O, Gorukmez O, Sag SO, Erdol S, Saglam H, Yakut T. NOVEL MUTATION OF THE ELECTRON TRANSFERRING FLAVOPROTEIN DEHYDROGENASE (ETFDH) GENE IN THE ISOLATED MYOPATHIC FORM OF COENZYME q10 DEFICIENCY. Genet Couns. 2015;26 2:259-62.

60. Fitzgerald M, Crushell E, Hickey C. Cyclic vomiting syndrome masking a fatal metabolic disease. European journal of pediatrics. 2013;172 5:707-10; doi: 10.1007/s00431-012-1852-z.

61. Scheicht D, Werthmann ML, Zeglam S, Holtmeier J, Holtmeier W, Strunk J. [Muscle weakness and early stages of liver failure in a 22-year-old man]. Der Internist. 2013;54 8:1016-22; doi: 10.1007/s00108-013-3329-1.

62. Rosenbohm A, Sussmuth SD, Kassubek J, Muller HP, Pontes C, Abicht A, et al. Novel ETFDH mutation and imaging findings in an adult with glutaric aciduria type II. Muscle Nerve. 2014;49 3:446-50; doi: 10.1002/mus.23979.

63. Lammer AB, Rolinski B, Ahting U, Heuss D. Multiple acyl-CoA-dehydrogenase deficiency (MADD)--a novel mutation of electron-transferring-flavoprotein dehydrogenase ETFDH. J Neurol Sci. 2011;307 1-2:166-7; doi: 10.1016/j.jns.2011.05.001.

64. Koppel S, Gottschalk J, Hoffmann GF, Waterham HR, Blobel H, Kolker S. Late-onset multiple acyl-CoA dehydrogenase deficiency: a frequently missed diagnosis? Neurology. 2006;67 8:1519; doi: 10.1212/01.wnl.0000240065.35635.a6.

65. Gempel K, Topaloglu H, Talim B, Schneiderat P, Schoser BG, Hans VH, et al. The myopathic form of coenzyme Q10 deficiency is caused by mutations in the electron-transferring-flavoprotein dehydrogenase (ETFDH) gene. Brain. 2007;130 Pt 8:2037-44; doi: 10.1093/brain/awm054.

66. Horvath R. Update on clinical aspects and treatment of selected vitamin-responsive disorders II (riboflavin and CoQ 10). J Inherit Metab Dis. 2012;35 4:679-87; doi: 10.1007/s10545-011-9434-1.

67. Trakadis Y, Kadlubowska D, Barnes R, Mitchell J, Spector E, Frerman F, et al. Pregnancy of a patient with multiple Acyl-CoA dehydrogenation deficiency (MADD). Mol Genet Metab. 2012;106 4:491-4; doi: 10.1016/j.ymgme.2012.05.001.

68. Olsen RK, Andresen BS, Christensen E, Bross P, Skovby F, Gregersen N. Clear relationship between ETF/ETFDH genotype and phenotype in patients with multiple acyl-CoA dehydrogenation deficiency. Hum Mutat. 2003;22 1:12-23; doi: 10.1002/humu.10226.

69. Beresford MW, Pourfarzam M, Turnbull DM, Davidson JE. So doctor, what exactly is wrong with my muscles? Glutaric aciduria type II presenting in a teenager. Neuromuscul Disord. 2006;16 4:269-73; doi: 10.1016/j.nmd.2006.01.001.

70. Olsen RK, Olpin SE, Andresen BS, Miedzybrodzka ZH, Pourfarzam M, Merinero B, et al. ETFDH mutations as a major cause of riboflavin-responsive multiple acyl-CoA dehydrogenation deficiency. Brain. 2007;130 Pt 8:2045-54; doi: 10.1093/brain/awm135.

71. Gregersen N, Wintzensen H, Christensen SK, Christensen MF, Brandt NJ, Rasmussen K. C6-C10-dicarboxylic aciduria: investigations of a patient with riboflavin responsive multiple acyl-CoA dehydrogenation defects. Pediatr Res. 1982;16 10:861-8; doi: 10.1203/00006450-198210000-00012.

72. Gregersen N. Riboflavin-responsive defects of beta-oxidation. J Inherit Metab Dis. 1985;8 Suppl 1:65-9.

73. Stojanovic N, Walker V, Gatling W, Coppini DV. MADD or drunk? Adults have inborn errors too. Hospital medicine (London, England : 1998). 2000;61 3:212-3.

74. Olsen RK, Andresen BS, Christensen E, Mandel H, Skovby F, Nielsen JP, et al. DNA-based prenatal diagnosis for severe and variant forms of multiple acyl-CoA dehydrogenation deficiency. Prenat Diagn. 2005;25 1:60-4; doi: 10.1002/pd.983.
